# Supplementary material for: Ketamine’s acute effects on negative brain states are mediated through distinct altered states of consciousness in humans
Source: Nat Commun. 2023 Oct 19;14:6631. doi: 10.1038/s41467-023-42141-5 (PMC10587184; doi:10.1038/s41467-023-42141-5)
Supplement: Supplementary file 1 — Supplementary Information [file 41467_2023_42141_MOESM1_ESM.pdf]

Ketamine's acute effects on negative brain states are mediated through distinct altered states of consciousness in humans

Laura M. Hack, Xue Zhang, Boris D. Heifets, Trisha Suppes, Peter J. van Roessel, Jerome A. Yesavage, Nancy J. Gray, Rachel Hilton, Claire Bertrand, Carolyn I. Rodriguez, Karl Deisseroth, Brian Knutson, & Leanne M. Williams

Supplementary Information

SUPPLEMENTARY METHODS

Participants

Participants were recruited through Facebook Ads using Institutional Review Board-approved material. Individuals who expressed interest in the study were directed to an online screening survey in REDCap. Individuals who were eligible to participate were contacted by a research coordinator for a telephone screening. On this telephone call, research coordinators provided the individuals with additional information about the study, obtained informed consent, collected additional demographic information, and scheduled an in-person screening visit at a research clinic. Screening procedures were conducted by trained medical professionals including phlebotomists (blood draw), research nurses (ECG and vital signs), trained research coordinators (drug and psychiatric histories), and licensed study physicians (physical examinations and confirmation of psychiatric histories). Baseline and infusion session procedures were conducted by trained research coordinators (study assessments), research nurses (peripheral IV catheter placement), and licensed study physicians (ketamine infusions, monitoring, and safety assessments). Travel was arranged for participants to all infusion visits, and they were financially compensated for their participation in the study. We aimed to and were able to recruit equal

numbers of males and females to control for the self-reported biological sex of participants. We included biological sex as a confounder for linear mixed models that were used to examine ketamine-induced dose-dependent effects. However, we do acknowledge with our current sample size we might be under-powered to examine sex differences in the acute impact of ketamine on human neural circuit function.

Assessments of dissociation and other ASCs

<u>Addressing data missingness</u>

For each subject's questionnaire data under a certain dose condition, if the missing items were fewer than 10% of the total item numbers of the questionnaire, we replaced missing items with the group mean of that dose condition. This brought the sample size of CADSS to be n = 13, n = 12, n = 13 for placebo, 0.05 mg/kg, and 0.5 mg/kg and of 5D-ASC to be n = 13 for all three drug visits.

Linear mixed effects and t-test analyses of dose-dependent effects of ketamine on dissociation and other ASCs

For Clinician-Administered Dissociative States Scale (CADSS) subscales assessed at T = 0 min and 40 mins, the linear mixed effects model (LMM) included dose (placebo, 0.05 mg/kg, or 0.5 mg/kg), time (T = 0 min and 40 mins), and the dose-by-time interaction as fixed effects and subject as the random effect. For 5-Dimensional Altered States of Consciousness (5D-ASC) subscales collected only post-drug administration, the LMM included only dose as the fixed effect and subject as the random effect. For post-hoc paired t-tests between drug dose conditions and for later mediation analysis, we derived the post- versus pre-infusion change of each CADSS subcomponent.

Brain imaging

<u>Structural Magnetic Resonance Imaging (MRI)</u>

A T1-weighted and a T2-weighted anatomical MRI image were collected at the baseline visit for normalization of all functional MRI data into standard space. The T1-weighted MRI was collected in sagittal orientation with TR = 3.0 s, TE = 3.828 ms, FA = 8°, acquisition time = 8:33, field of view (FOV) = 256 × 256 mm, 3D matrix size = 320 × 320 × 230, voxel size = 0.8 mm isotropic, motion correction = PROMO. The T2-weighted MRI was collected in sagittal orientation with TR = 2.5 s, TE = Maximum, FA = 90, acquisition time = 5:42, FOV = 240 × 240 mm, 3D matrix size = 320 × 320 × 216, voxel size = 0.8 mm isotropic, motion correction = PROMO.

Image Preprocessing

Results included in this paper come from preprocessing performed using fMRIPrep 20.2.3[4,5] (RRID:SCR_016216), which is based on Nipype 1.6.1[6,7]; RRID:SCR_002502).

Anatomical Data Preprocessing

A total of 1 T1-weighted (T1w) image found within the input BIDS dataset. The T1-weighted (T1w) image was corrected for intensity non-uniformity (INU) with N4BiasFieldCorrection[8], distributed with ANTs 2.3.3[9] (RRID:SCR_004757), and used as T1w-reference throughout the workflow. The T1w-reference was then skull-stripped with a Nipype implementation of the antsBrainExtraction.sh workflow (from ANTs), using OASIS30ANTs as target template. Brain tissue segmentation of cerebrospinal fluid (CSF), white-matter (WM) and gray-matter (GM) was performed on the brain-extracted T1w using fast (FSL 5.0.9, RRID:SCR_002823[10]). Brain surfaces were reconstructed using recon-all (FreeSurfer 6.0.1, RRID:SCR_001847[11]) and the brain mask estimated previously was refined with a custom variation of the method to reconcile ANTs-derived and FreeSurfer-derived segmentations of the cortical gray-matter of Mindboggle (RRID:SCR_002438[12]). Volume-based spatial normalization to two standard spaces (MNI152Nlin6Asym, MNI152Nlin2009cAsym) was performed through nonlinear registration with antsRegistration (ANTs 2.3.3), using brain-extracted versions of both T1w reference and the T1w

template. The following templates were selected for spatial normalization: FSL's MNI ICBM 152 non-linear 6th Generation Asymmetric Average Brain Stereotaxic Registration Model[13] [RRID:SCR_002823; TemplateFlow ID: MNI152NLin6Asym], ICBM 152 Nonlinear Asymmetrical template version 2009c [[14], RRID:SCR_008796; TemplateFlow ID: MNI152NLin2009cAsym].

Functional Data Preprocessing

For each of the BOLD runs found per subject (across all tasks and sessions), the following preprocessing was performed. First, a reference volume and its skull-stripped version were generated by aligning and averaging 1 single-band references (SBRefs). Susceptibility distortion correction (SDC) was omitted. The BOLD reference was then co-registered to the T1w reference using bbregister (FreeSurfer) which implements boundary-based registration[15]. Co-registration was configured with six degrees of freedom. Head-motion parameters with respect to the BOLD reference (transformation matrices, and six corresponding rotation and translation parameters) are estimated before any spatiotemporal filtering using mcflirt (FSL 5.0.9[16]). First, a reference volume and its skull-stripped version were generated using a custom methodology of fMRIPrep. The BOLD time-series were resampled onto the following surfaces (FreeSurfer reconstruction nomenclature): fsnative, fsaverage. The BOLD time-series (including slice-timing correction when applied) were resampled onto their original, native space by applying the transforms to correct for head-motion. These resampled BOLD time-series will be referred to as preprocessed BOLD in original space, or just preprocessed BOLD. The BOLD time-series were resampled into several standard spaces, correspondingly generating the following spatially normalized, preprocessed BOLD runs: MNI152NLin6Asym, MNI152NLin2009cAsym. First, a reference volume and its skull-stripped version were generated using a custom methodology of fMRIPrep. Grayordinates files[17] containing 91k samples were also generated using the highest resolution fsaverage as intermediate standardized surface space. Automatic removal of motion artifacts using independent component analysis (ICA-AROMA,[18]) was performed on the preprocessed BOLD on

MNI space time-series after removal of non-steady state volumes and spatial smoothing with an isotropic, Gaussian kernel of 6mm FWHM (full-width half-maximum). Corresponding "non-aggressively" denoised runs were produced after such smoothing. Additionally, the "aggressive" noise-regressors were collected and placed in the corresponding confounds file. Several confounding time-series were calculated based on the preprocessed BOLD: framewise displacement (FD), DVARS and three region-wise global signals. FD was computed using two formulations following Power (absolute sum of relative motions[19]) and Jenkinson (relative root mean square displacement between affines[16]). FD and DVARS are calculated for each functional run, both using their implementations in Nipype (following the definitions by[19]). The three global signals are extracted within the CSF, the WM, and the whole-brain masks. Additionally, a set of physiological regressors were extracted to allow for component-based noise correction (CompCor[20]). Principal components are estimated after high-pass filtering the preprocessed BOLD time-series (using a discrete cosine filter with 128s cut-off) for the two CompCor variants: temporal (tCompCor) and anatomical (aCompCor). tCompCor components are then calculated from the top 2% variable voxels within the brain mask. For aCompCor, three probabilistic masks (CSF, WM, and combined CSF+WM) are generated in anatomical space. The implementation differs from that of Behzadi et al. in that instead of eroding the masks by 2 pixels on BOLD space, the aCompCor masks are subtracted a mask of pixels that likely contain a volume fraction of GM. This mask is obtained by dilating a GM mask extracted from the FreeSurfer's aseg segmentation, and it ensures components are not extracted from voxels containing a minimal fraction of GM. Finally, these masks are resampled into BOLD space and binarized by thresholding at 0.99 (as in the original implementation). Components are also calculated separately within the WM and CSF masks. For each CompCor decomposition, the k components with the largest singular values are retained, such that the retained components' time series are sufficient to explain 50 percent of variance across the nuisance mask (CSF, WM, combined, or temporal). The remaining components are dropped from consideration. The head-motion estimates calculated in the

correction step were also placed within the corresponding confounds file. The confound time series derived from head motion estimates and global signals were expanded with the inclusion of temporal derivatives and quadratic terms for each[21]. Frames that exceeded a threshold of 0.5 mm FD or 1.5 standardized DVARS were annotated as motion outliers. All resampling's can be performed with a single interpolation step by composing all the pertinent transformations (i.e., head-motion transform matrices, susceptibility distortion correction when available, and co-registrations to anatomical and output spaces). Gridded (volumetric) resampling was performed using antsApplyTransforms (ANTs), configured with Lanczos interpolation to minimize the smoothing effects of other kernels[22]. Non-gridded (surface) resampling was performed using mri_vol2surf (FreeSurfer).

Many internal operations of fMRIPrep use Nilearn 0.6.2[23] (RRID:SCR_001362), mostly within the functional processing workflow. For more details of the pipeline, see the section corresponding to workflows in fMRIPrep's documentation.

Copyright Waiver

The above boilerplate text was automatically generated by fMRIPrep with the express intention that users should copy and paste this text into their manuscripts unchanged. It is released under the CC0 license. The boilerplate material begins at the section titled: Image Preprocessing.

Quality control procedure

Quality control diagnostics included visual inspection of the raw fMRI timeseries for artifacts and signal dropout, and a review of the fMRIprep summary report for each participant. Participants' data were excluded if more than 25% (37/148) of time points were detected as motion spikes. Volumes with frame-wise displacement >0.5 mm or std DVARS >1.5 are defined as motion spikes. One participant's data for the 0.05 mg/kg was excluded. One participant was not able to

complete brain scans due to nausea under the 0.5 mg/kg condition. One participant was unreachable after the completion of the first two scan visits (placebo and 0.5 mg/kg) and is missing the 0.05 mg/kg data. This resulted in n = 13, 11, and 12 for placebo, 0.05mg/kg, and 0.5mg/kg conditions, respectively.

Extracting behavioral performance

Accuracy, measured by number of button presses with reaction time (RT) > 200ms, and the mean RT of correct presses, were extracted from each subject's behavior data recorded from a button box.

Facial Expression of Emotion Task (FEET) data analysis of dose-dependent effects of ketamine on behavioral performance

To examine dose-dependent effects of ketamine on individuals' behavioral performance during FEET, we used linear mixed effects models (LMMs) with accuracy or mean RT as the dependent variable and with dose (placebo, 0.05 mg/kg, or 0.5 mg/kg) as the fixed effect.

Definition of region of interest (ROI)

As established in our previous work[24], we defined ROIs with an automated meta-analysis approach using neurosynth.org. Specifically, we used Neurosynth uniformity (previously called forward inference) maps with a false discovery rate (FDR) threshold of .01 for the search terms of anterior insula, amygdala, subgenual anterior cingulate cortex (sgACC), and dorsal anterior cingulate cortex (dACC). We imposed a restriction that the peak of the ROIs should have a minimum z-score of 6. For the anterior insula, we also excluded voxels with a z-score <5 to keep only the most relevant voxels spatially located in the anterior portion of the insula via visual inspection. For the amygdala, Neurosynth maps were restricted by anatomically defined boundaries from the Automated Anatomical Labeling atlas.

Sensitivity analysis of dose-dependent effects of ketamine on anterior insula and amygdala activity in response to threat confounded by behavioral performance

As additional sensitivity analyses, we added the accuracy (button press number) and mean RT as covariates of no interest when examining ketamine's dose-dependent effect on FEET activations in selected ROIs.

Sensitivity analysis of dose-dependent effects of ketamine on dissociation and altered states of consciousness, and on anterior insula and amygdala activity in response to threat after controlling for the order of drug visits

To test whether the order of drug visits had an impact on our current finding, we created a categorical variable representing the six drug visit orders: i) placebo, 0.05 mg/kg, 0.5 mg/kg; ii) placebo, 0.5 mg/kg, 0.05 mg/kg; iii) 0.05 mg/kg, placebo, 0.5 mg/kg; iv) 0.05 mg/kg, 0.5 mg/kg, placebo; v) 0.5 mg/kg, placebo, 0.05 mg/kg; and vi) 0.5 mg/kg, 0.05 mg/kg, placebo. As another set of sensitivity analyses, we added the categorical order variable to all linear mixed models used for testing ketamine induced dose-dependent effects.

Mediation analysis of heart rate and blood pressure on ketamine's effects on amygdala and insula activity in response to threat

We monitored vital signs, including heart rate and systolic and diastolic blood pressure throughout each study visit. As an exploratory analysis, we also examined whether they mediated ketamine's effects on amygdala and insula activity in response to threat.

Intra-subject correlation analysis of subcomponents of dissociation and other altered states of consciousness

To explore the direct association between dissociation and other altered states of consciousness, repeated-measures correlation analyses were run using the rmcorr package (https://cran.r-project.org/package=rmcorr). Unlike conventional inter-subject correlation analysis, the repeated measures correlation analysis calculates intra-subject correlations, which in our case, reflects how different subcomponents of dissociation and other altered states of consciousness co-vary across visits. As with the mediation analysis, we did not include the 0.05 mg/kg condition in the correlation analysis because no significant change was observed for this condition compared to placebo. FDR correction was implemented to control for false positives.

SUPPLEMENTARY RESULTS

Ketamine does not alter behavioral performance during the FEET task

No significant dose-dependent change was detected for neutral, threat, or threat vs neutral conditions. These data are visualized in Suppl. Fig. 3.

Ketamine's effect on insula and amygdala activity remains the same when controlling for behavior performance

In additional sensitivity analyses controlling for button presses and mean RT, we similarly observed dose-dependent effects of ketamine on the activity of right anterior insula ($F_{2,22}$ = 9.14, p = 0.001) and right amygdala ($F_{2,22}$ = 6.68, p = 0.005) evoked by threat faces.

Ketamine's effect on altered states of consciousness and dissociation, and on anterior insula and amygdala activity in response to threat remains the same when controlling for the order of drug visits

We still observed a significant dose-dependent effect of ketamine in dissociative depersonalization ($F_{2,76}$ = 11.10, p < 0.001), dissociative derealization ($F_{2,76}$ = 19.27, p < 0.001), dissociative amnesia ($F_{2,76}$ = 7.57, p = 0.001), altered states of bliss ($F_{2,25}$ = 8.66, p = 0.001), anxiety ($F_{2,25}$ = 36.57, p < 0.001), and impaired control and cognition ($F_{2,25}$ = 31.75, p < 0.001). Similarly, we observed dose-dependent effects of ketamine on increasing right anterior insula ($F_{2,36}$ = 9.23, p < 0.001) and right amygdala activity ($F_{2,19}$ = 18.46, p < 0.001) evoked by threat faces.

Neither heart rate or blood pressures mediates ketamine's effects on amygdala and insula activity in response to threat

No significant mediating effect was found for heart rate or blood pressure measures (**Suppl. Table 3**).

Intra-correlation of subcomponents of dissociation and other altered states of consciousness

Although the dissociation subcomponents depersonalization and amnesia were correlated with derealization (Repeated Measures Correlation; depersonalization to derealization r = 0.93, pFDR < 0.001; amnesia to derealization r = 0.66, pFDR = 0.05), they were not correlated with each other (Repeated Measures Correlation r = 0.52, pFDR = 0.13), indicating that they may map onto different factors of ketamine-related experiences. As expected, greater amnesia was associated with greater fear/anxiety, measured by the 5-Dimensional Altered States of Consciousness (5D-ASC) rating scale (Repeated Measures Correlation r = 0.75, pFDR = 0.02).

Suppl. Table 1. Comparison of post-hoc paired t-test results between the placebo, 0.05 mg/kg and 0.5 mg/kg conditions

Measures		Placebo	0.05 mg/kg	0.5 mg/kg	0.05 mg/kg vs. Placebo			0.5 mg/kg vs. Placebo			0.5 mg/kg vs. 0.05 mg/kg		
		Mean (SD)	Mean (SD)	Mean (SD)	T	p	*d*	T	p	*d*	T	p	*d*
ASCs Hypothesized to Relieve Negative Affective Brain States	Depersonalization	0 (0)	0.52 (1.22)	13.22 (16.69)	1.48	0.17	0.45	2.85	**0.02***	0.82	2.87	**0.02***	0.86
	Derealization	0.67 (1.30)	0.69 (1.36)	14.72 (13.89)	-0.05	0.96	0.02	3.69	**0.003****	1.06	3.59	**0.004****	1.08
	Bliss	4.59 (13.27)	6.11 (9.41)	25 (28.13)	0.39	0.70	0.12	2.62	**0.02***	0.76	2.27	**0.04***	0.68
ASCs Hypothesized to Exacerbate Negative Affective Brain States	Amnesia	0 (0)	1.39 (3.24)	9.03 (13.01)	1.48	0.17	0.45	2.50	**0.03***	0.72	1.97	0.07	0.60
	Anxiety	2.04 (4.57)	2.57 (4.66)	15.38 (11.00)	1.19	0.26	0.36	5.30	**<0.001*****	1.53	4.94	**<0.001*****	1.49
	Impaired control and cognition	2.46 (4.58)	2.89 (5.67)	23.56 (15.05)	0.38	0.71	0.12	5.24	**<0.001*****	1.51	4.86	**<0.001*****	1.47
Neural activity in response to threat versus neutral	Right anterior insula	-0.06 (0.68)	-0.28 (0.33)	0.56 (0.32)	-0.49	0.64	0.27	3.22	**0.009****	1.02	6.50	**<0.001*****	2.30
	Right amygdala	-0.29 (0.78)	0.11 (0.48)	0.68 (0.50)	1.56	0.15	0.48	4.05	**0.002****	1.28	3.30	**0.009****	0.92

Abbreviations: ASCs = Altered States of Consciousness; SD = standard deviation; T = T score from paired t-tests; p = p value from paired t-tests; *d* = Cohen's *d* effect size from pairwise contrasts; *: $p < 0.05$; **: $p < 0.01$; ***: $p < 0.001$. Paired t-tests were two-sided. Source data are provided as a Source Data file.

Suppl. Table 2. Summary of individuals' altered states of consciousness scores and their quotes at 20-30 minutes into the infusion

Subjects	Depersonalization	Derealization	Bliss	Amnesia	Anxiety	Impaired control and cognition	Quotes
**P09**	18.8	12.5	0	8.3	10.3	2.1	*"Feeling dissociated and spaced out. Feeling tingly, numb, spacey. Do I feel lighter? Is my hand heavier or lighter? I can't tell if my head is farther or closer to the ground."*
**P10**	53.1	41.7	25.3	0	-1.7	7.7	*"I feel like I'm floating. I feel like I'm in a spaceship. Super disoriented- there is no north or south. I'm in the center and there's no bottom. Geometric shapes are felt not seen. It's not like a euphoria per se, but it feels very expansive, yet restrictive at the same time. Feels like I'm in a tunnel. Stretchy wobbly sensation. Space is stretching out and coming back."*
**P11**	3.1	10.4	2	8.3	13.8	41.8	*"I'm feeling weird, my body is tingling, hearing a hum, especially in my right ear. I've never felt this way before, neutral color all around. Chair legs, there is other stuff around but can only focus on the chair. It feels like there is a foam spray insulation around me, I feel like I can see my thoughts from a distant perspective."*
**P12**	3.1	15.2	-0.7	0	17.3	21.6	*"I felt spaced out at 10 minutes. I felt blurry, time felt stretched out. When I close my eyes, it feels like it's going to shake. I felt a little bit stoned, but with 'annoying' pot. I'm aware of heartbeat and feeling spaced out. Felt disconnected from body, time felt stretched and that it would take time to do anything."*
**P13**	6.3	7.5	0	25.7	20.2	33	*"I feel like a 2D character."*

Each measure is quantified as the change induced by ketamine at 0.5 mg/kg relative to placebo condition. Source data are provided as a Source Data file.

Suppl. Table 3. Summary of results from mediation analysis

DV		Mediator	ACME		ADE		Total Effect	
			Coef	p-value	Coef	p-value	Coef	p-value
Neural activity in response to threat versus neutral	Right anterior insula	Depersonalization	**-0.39**	**0.004****	1.52	<0.001	1.13	<0.001
		Derealization	-0.22	0.3	1.2	<0.001	0.99	<0.001
		Bliss	0.13	0.41	0.86	<0.001	0.98	<0.001
		Amnesia	**0.32**	**0.04***	0.65	0.01	1.00	<0.001
		Anxiety	**0.46**	**0.08⁺**	0.69	0.07	0.99	<0.001
		Impaired control and cognition	0.28	0.3	0.7	0.04	0.98	<0.001
		Heart rate	-0.02	0.97	0.84	0.01	0.83	<0.001
		SBP	0.14	0.18	0.68	<0.001	0.82	<0.001
		DBP	0.14	0.18	0.68	<0.001	0.82	<0.001
	Right amygdala	Depersonalization	0.04	0.85	1.06	<0.001	1.13	<0.001
		Derealization	0.27	0.12	0.82	<0.001	0.99	<0.001
		Bliss	0.16	0.19	0.93	<0.001	0.98	<0.001
		Amnesia	0.23	0.10	0.86	<0.001	1.00	<0.001
		Anxiety	0.21	0.39	0.89	<0.001	0.99	<0.001
		Impaired control and cognition	0.29	0.25	0.71	<0.001	0.98	<0.001
		Heart rate	-0.06	0.74	0.99	<0.001	0.83	<0.001
		SBP	0.04	0.5	0.88	<0.001	0.82	<0.001
		DBP	0.01	0.86	0.91	<0.001	0.82	<0.001

Abbreviations: DV = Dependent Variable; ACME = Average Causal Mediation Effects; ADE = Average Direct Effects; SBP = systolic blood pressure; DBP = diastolic blood pressure; Coef = partially standardized coefficient estimated; $^{+}$: $p < 0.1$; *: $p < 0.05$; **: $p < 0.01$. Source data are provided as a Source Data file.

The standardized beta coefficient values of the ACME reflect the effect size of the mediation, in our case indicating ketamine at 0.5 mg/kg dose induced change of neural activity in the magnitude of standard deviation (SD) through the mediating effect of dissociation and other ASCs.

Suppl. Table 4. Inclusion and exclusion criteria

**Inclusion Criteria**	Ages 18-55
	All genders and ethno-racial categories
	Able and willing to enroll and provide written informed consent
	Able to comply with study procedures
	Able to receive an MRI
	At least 2 prior uses of ketamine when aged 18 years or older
**Exclusion Criteria**	Prior adverse ketamine response
	Use of ketamine in past 7 days
	Current mood, anxiety, eating, or psychotic disorder
	Allergy or hypersensitivity to ketamine
	Current use of psychotropic medication. If participants are currently on psychotropic medications, a washout period of 5 half-lives will be required prior to the infusion visits.
	Schizophrenia in a first degree relative
	MRI contraindication
	Concurrent use of any medications that might increase the risk of participation (e.g., drug interactions)
	Cannabis use in the past 7 days, alcohol use in the past 24 hours, and/or other illicit recreational drug use in the 48 hours prior to sessions. In addition to self-report, these exclusion criteria will be assessed by a urine screen, saliva sample, or breathalyzer.
	Renal/hepatic impairment
	Hypertension (Hypertension, Stage 1 as defined by a systolic blood pressure >140 mmHg or diastolic blood pressure > 90 mmHg on two of three measurements at least 15 minutes apart at initial screening appointment; systolic blood pressure >155 mmHg or diastolic blood pressure >99 mmHg on two of three measurements at least 15 minutes apart during infusion visits).
	Heart rate <50bpm or >150bpm assessed at initial screening visit
	Chronic congestive heart failure, tachyarrhythmias, myocardial ischemia (assessed via EKG at initial screening appointment)
	EKG QTcF intervals >430msec for men and >470msec for women
	History of epilepsy, convulsions, seizures, loss of consciousness > 10 min assessed at screening

Abbreviations: EKG = Electrocardiogram; MRI = Magnetic Resonance Imaging.

Suppl. Table 5. Demographics and baseline clinical depression and anxiety levels

Features	Overall (N=13)
**Age (years)**	
Mean (SD)	33.3 (9.82)
Median [Min, Max]	35.0 [22.0, 51.0]
**Gender**	
Male	6 (46.2%)
Female	7 (53.8%)
**Hamilton Anxiety Scale**	
Mean (SD)	0.769 (1.17)
Median [Min, Max]	0 [0, 4.00]
**Hamilton Depression Scale**	
Mean (SD)	0.308 (0.480)
Median [Min, Max]	0 [0, 1.00]
**9-item Patient Health Questionnaire**	
Mean (SD)	2.15 (3.21)
Median [Min, Max]	1.00 [0, 12.0]
**7-Item Generalized Anxiety Disorder**	
Mean (SD)	1.23 (1.09)
Median [Min, Max]	1.00 [0, 3.00]

Suppl. Figure 1. Outcomes regarding altered states of consciousness (ASCs) hypothesized to associate with negative affective brain states.

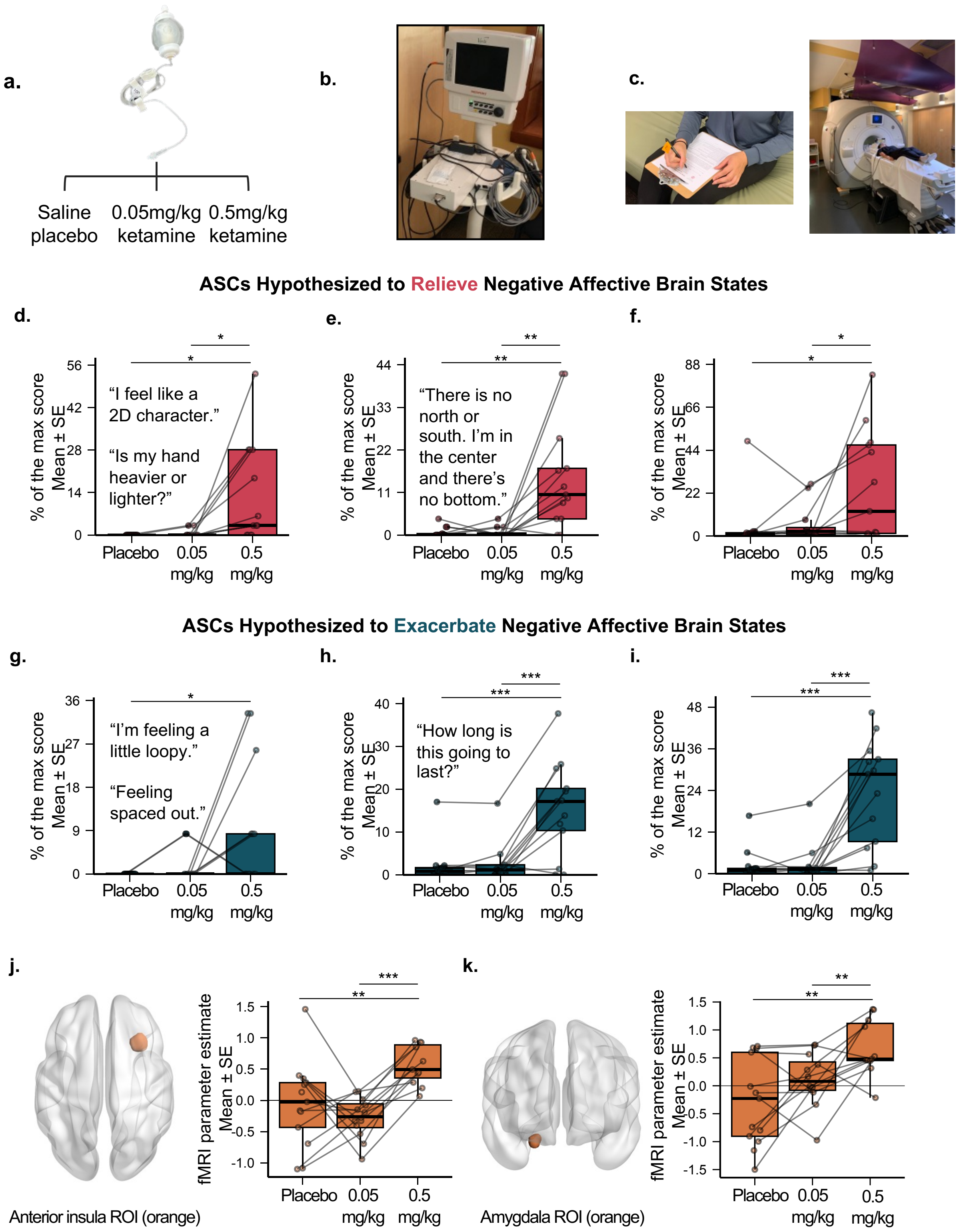

a. Randomized dose (X) (n=13 nonclinical participants). **b.** Licensed study clinicians monitored heart rate, blood pressure, and pulse oximetry during infusion and scan with

an MRI-safe machine. **c.** Multi-modal measures were collected prior to and after infusion. Permission was obtained from the individuals shown in the photographs for use of their images. Several sub-figures show the results of comparing outcomes between the three drug conditions with regard to **d.** dissociative depersonalization, **e.** dissociative derealization, **f.** altered states of bliss, **g.** dissociative amnesia, **h.** altered states of anxiety, and **i.** impaired control and cognition. Participants' quotes related to specific subcomponents of dissociation and altered states of consciousness are shown within these sub-figures. **j.** Right anterior insula activity in response to threat faces. **k.** Right amygdala activity in response to threat faces. One-way repeated Analysis of Variance was used to analyze the data. Adjustments for multiple comparisons were made. In each boxplot, the central thick black line represents the median, color shaded boxes represent the first and third quartiles (the 25th and 75th percentiles), and the whiskers extend no further than 1.5 times of the distance between the first and third quartiles. *: $p < 0.05$, **: $p < 0.01$, ***: $p < 0.001$. Source data are provided as a Source Data file.

Suppl. Figure 2. Nonconscious Facial Expression of Emotion Task (FEET)

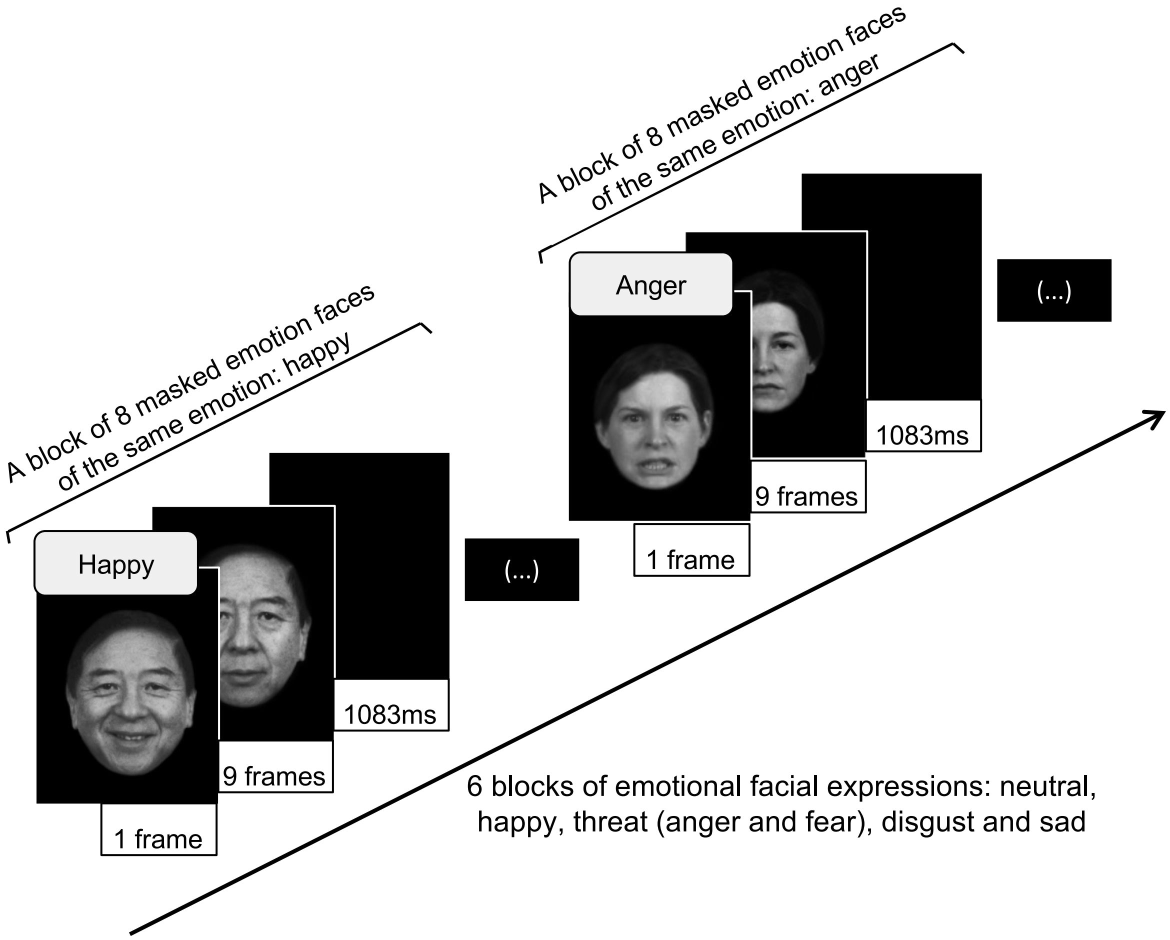

A total of 240 standardized facial expressions from 6 different emotions (neutral, happy, threat [anger and fear], disgust, and sad) were presented in emotional blocks, with each block consisting of 8 faces of the same emotion and each block repeated for 5 times in a pseudorandom order. For each trial within a block, an emotional face was presented for 16.7 ms (1 monitor frame), followed immediately by a neutral face perceptual mask for 150.3 ms (9 monitor frames) and an interstimulus interval of 1083 ms. The facial expressions were extracted from Figure 1 of Williams et al., 2015[25]. These images were modified with permission from the developers of a database of 3D facial stimuli described in Gur et al., 2002[26.]

Suppl. Figure 3. Behavioral performances during the nonconscious Facial Expression of Emotion Task (FEET)

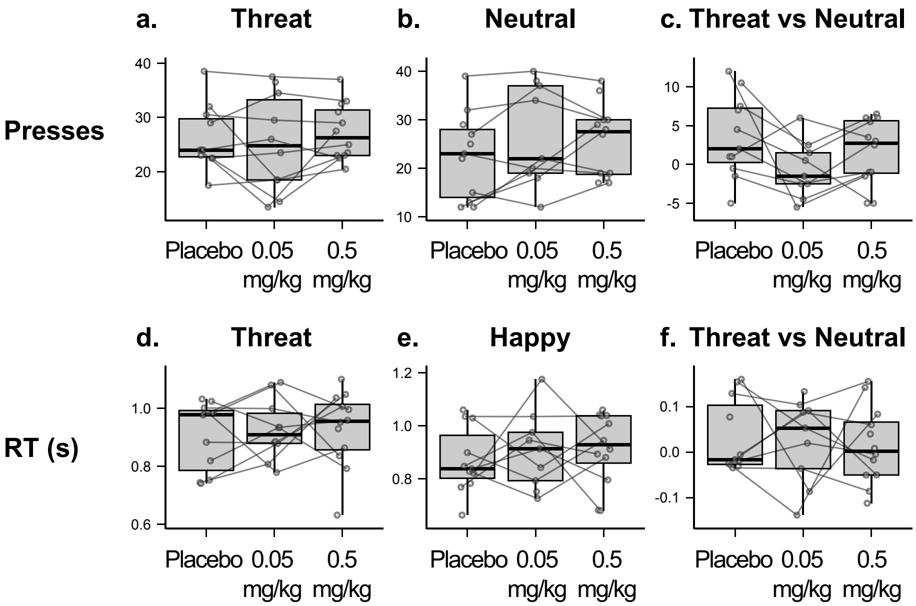

Accuracy (number of correct presses) for n=13 nonclinical participants under Threat (**a.**), Neutral (**b**.), and Threat vs Neutral (**c**.). Average reaction time (RT) of the correct presses under Threat (**d**.), Neutral (**e**.), and Threat vs Neutral (**f**.). One-way repeated Analysis of Variance was used to analyze the data. In each boxplot, the central thick black line represents the median, color shaded boxes represent the first and third quartiles (the 25th and 75th percentiles), and the whiskers extend no further than 1.5 times of the distance between the first and third quartiles. Source data are provided as a Source Data file.

Suppl. Figure 4. Experiences of anxiety mediate distinct ketamine induced increases in insula activation in response to social threat

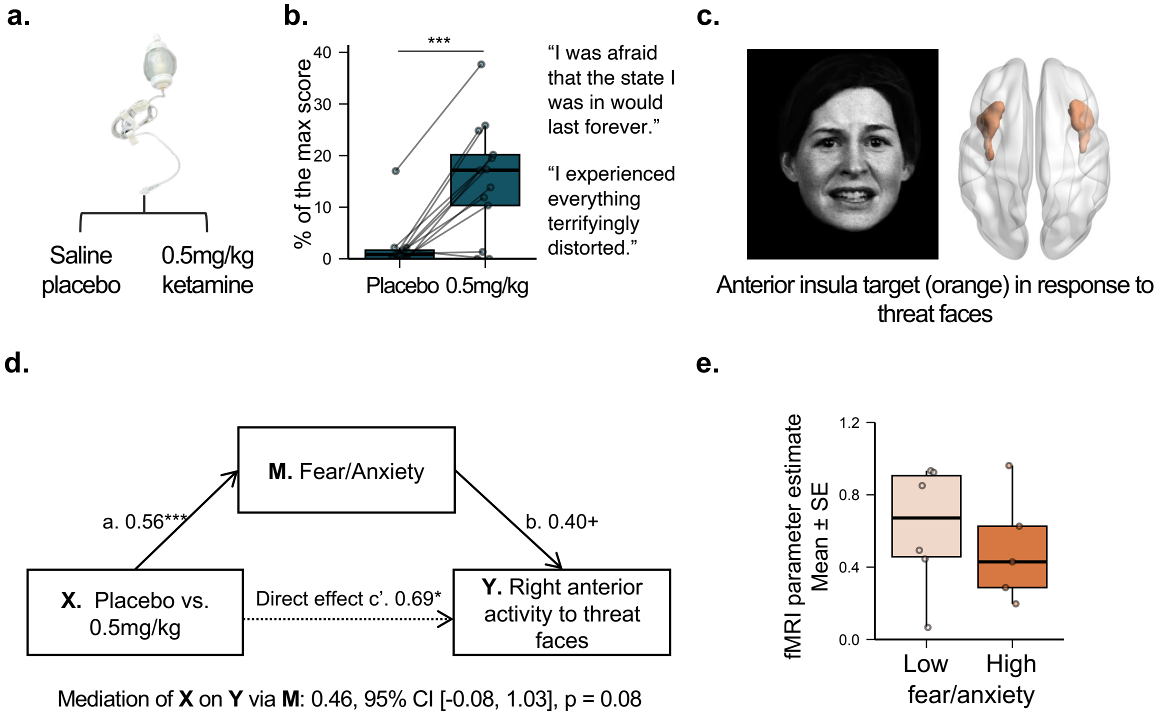

a. Randomized dose (**X**) (n=13 clinical participants). **b.** Altered states of anxiety (**M**) at the end of infusion (40 minutes). Example items are listed. A two-sided post-hoc paired t-test was used to compare the effect of placebo vs. 0.5mg/kg of ketamine on anxiety (T_{12} = 5.30, p < 0.001, Cohen's d = 1.53, 95% CI = [7.85, 18.82]). We implemented an FDR correction to control for the testing of multiple scale sub-components. **c.** Anterior insula engaged by threat faces (**Y**). The facial expression was extracted from Figure 1 of Williams et al., 2015[25]. The image was modified with permission from the developers of a database of 3D facial stimuli described in Gur et al., 2002[26]. **d.** Ketamine-induced effects (**X**) on reducing right anterior insula activity to threat faces (**Y**). An Averaged Causal Mediation Effect (ACME) mediation model was used to assess whether ketamine-induced anxiety mediates the effect of dose on acute changes in neural activity during emotional processing. No adjustments for multiple comparisons were made. **e.** Right anterior insula activity in response to threat faces as a function of fear/anxiety. A mediation model was used to analyze the data. Each bar graph displays the group mean and standard error (SE). +: p < 0.1, *: p <0.05, **: p < 0.01, ***: p<0.001. Source data are provided as a Source Data file. *Abbreviations:* fMRI = functional magnetic resonance imaging.

SUPPLEMENTARY REFERENCES

1 Scheidegger, M. *et al.* Ketamine administration reduces amygdalo-hippocampal reactivity to emotional stimulation. *Hum Brain Mapp* **37**, 1941-1952 (2016). https://doi.org:10.1002/hbm.23148

2 Nugent, A. C. *et al.* Ketamine has distinct electrophysiological and behavioral effects in depressed and healthy subjects. *Molecular Psychiatry* **24**, 1040-1052 (2019). https://doi.org:10.1038/s41380-018-0028-2

3 Faul, F., Erdfelder, E., Lang, A.-G. & Buchner, A. G*Power 3: A flexible statistical power analysis program for the social, behavioral, and biomedical sciences. *Behavior Research Methods* **39**, 175-191 (2007). https://doi.org:10.3758/BF03193146

4 Esteban, O. *et al.* fMRIPrep: a robust preprocessing pipeline for functional MRI. *Nat Methods* **16**, 111-116 (2019). https://doi.org:10.1038/s41592-018-0235-4

5 10.5281/zenodo.852659, f. A. f.

6 Gorgolewski, K. *et al.* Nipype: a flexible, lightweight and extensible neuroimaging data processing framework in python. *Front Neuroinform* **5**, 13 (2011). https://doi.org:10.3389/fninf.2011.00013

7 Gorgolewski, K. J. *et al.* Nipype: a flexible, lightweight and extensible neuroimaging data processing framework in Python. *Software. Zenodo.* (2018).

8 Tustison, N. J. *et al.* N4ITK: improved N3 bias correction. *IEEE Trans Med Imaging* **29**, 1310-1320 (2010). https://doi.org:10.1109/TMI.2010.2046908

9 Avants, B. B., Epstein, C. L., Grossman, M. & Gee, J. C. Symmetric diffeomorphic image registration with cross-correlation: evaluating automated labeling of elderly and neurodegenerative brain. *Med Image Anal* **12**, 26-41 (2008). https://doi.org:10.1016/j.media.2007.06.004

10 Zhang, Y., Brady, M. & Smith, S. Segmentation of brain MR images through a hidden Markov random field model and the expectation-maximization algorithm. *IEEE Trans Med Imaging* **20**, 45-57 (2001). https://doi.org:10.1109/42.906424

11 Dale, A. M., Fischl, B. & Sereno, M. I. Cortical surface-based analysis. I. Segmentation and surface reconstruction. *Neuroimage* **9**, 179-194 (1999). https://doi.org:10.1006/nimg.1998.0395

12 Klein, A. *et al.* Mindboggling morphometry of human brains. *PLoS Comput Biol* **13**, e1005350 (2017). https://doi.org:10.1371/journal.pcbi.1005350

13 Evans, A. C., Janke, A. L., Collins, D. L. & Baillet, S. Brain templates and atlases. *Neuroimage* **62**, 911-922 (2012). https://doi.org:10.1016/j.neuroimage.2012.01.024

14 Fonov, V. *et al.* Unbiased average age-appropriate atlases for pediatric studies. *Neuroimage* **54**, 313-327 (2011). https://doi.org:10.1016/j.neuroimage.2010.07.033

15 Greve, D. N. & Fischl, B. Accurate and robust brain image alignment using boundary-based registration. *NeuroImage* **48**, 63-72 (2009). https://doi.org:10.1016/j.neuroimage.2009.06.060

16 Jenkinson, M., Bannister, P., Brady, M. & Smith, S. Improved optimization for the robust and accurate linear registration and motion correction of brain images. *Neuroimage* **17**, 825-841 (2002). https://doi.org:10.1016/s1053-8119(02)91132-8

17 Glasser, M. F. *et al.* The minimal preprocessing pipelines for the Human Connectome Project. *Neuroimage* **80**, 105-124 (2013). https://doi.org:10.1016/j.neuroimage.2013.04.127

18 Pruim, R. H. R. *et al.* ICA-AROMA: A robust ICA-based strategy for removing motion artifacts from fMRI data. *Neuroimage* **112**, 267-277 (2015). https://doi.org:10.1016/j.neuroimage.2015.02.064

19 Power, J. D. *et al.* Methods to detect, characterize, and remove motion artifact in resting state fMRI. *Neuroimage* **84**, 320-341 (2014). https://doi.org:10.1016/j.neuroimage.2013.08.048
20 Behzadi, Y., Restom, K., Liau, J. & Liu, T. T. A component based noise correction method (CompCor) for BOLD and perfusion based fMRI. *Neuroimage* **37**, 90-101 (2007). https://doi.org:10.1016/j.neuroimage.2007.04.042
21 Satterthwaite, T. D. *et al.* An improved framework for confound regression and filtering for control of motion artifact in the preprocessing of resting-state functional connectivity data. *Neuroimage* **64**, 240-256 (2013). https://doi.org:10.1016/j.neuroimage.2012.08.052
22 Lanczos, C. Evaluation of Noisy Data. *Journal of the Society for Industrial and Applied Mathematics Series B Numerical Analysis 1* **1**, 76-85 (1964).
23 Abraham, A. *et al.* Machine learning for neuroimaging with scikit-learn. *Front Neuroinform* **8**, 14 (2014). https://doi.org:10.3389/fninf.2014.00014
24 Goldstein-Piekarski, A. N. *et al.* Mapping Neural Circuit Biotypes to Symptoms and Behavioral Dimensions of Depression and Anxiety. *Biol Psychiatry* **91**, 561-571 (2022). https://doi.org:10.1016/j.biopsych.2021.06.024
25 Williams, L. M. *et al.* Amygdala Reactivity to Emotional Faces in the Prediction of General and Medication-Specific Responses to Antidepressant Treatment in the Randomized iSPOT-D Trial. *Neuropsychopharmacology* **40,** 2398-408 (2015). https://doi.org:10.1038/npp.2015.89
26 Gur, R.C.*, et al.* A method for obtaining 3-dimensional facial expressions and its standardization for use in neurocognitive studies. *J Neurosci Methods* **115**, 137-143 (2002). https://doi.org:10.1016/s0165-0270(02)00006-7
