# Supplementary material for: Ketamine’s acute effects on negative brain states are mediated through distinct altered states of consciousness in humans
Source: Nat Commun. 2023 Oct 19;14:6631. doi: 10.1038/s41467-023-42141-5 (PMC10587184; doi:10.1038/s41467-023-42141-5)
Supplement: Supplementary file 3 — Reporting Summary [file 41467_2023_42141_MOESM3_ESM.pdf]

nature portfolio

Corresponding author(s): Leanne M. Williams

Last updated by author(s): 08/27/2023

Reporting Summary

Nature Portfolio wishes to improve the reproducibility of the work that we publish. This form provides structure for consistency and transparency in reporting. For further information on Nature Portfolio policies, see our Editorial Policies and the Editorial Policy Checklist.

Statistics

For all statistical analyses, confirm that the following items are present in the figure legend, table legend, main text, or Methods section.

n/a	Confirmed	
☐	☒	The exact sample size (*n*) for each experimental group/condition, given as a discrete number and unit of measurement
☐	☒	A statement on whether measurements were taken from distinct samples or whether the same sample was measured repeatedly
☐	☒	The statistical test(s) used AND whether they are one- or two-sided<br>*Only common tests should be described solely by name; describe more complex techniques in the Methods section.*
☐	☒	A description of all covariates tested
☐	☒	A description of any assumptions or corrections, such as tests of normality and adjustment for multiple comparisons
☐	☒	A full description of the statistical parameters including central tendency (e.g. means) or other basic estimates (e.g. regression coefficient) AND variation (e.g. standard deviation) or associated estimates of uncertainty (e.g. confidence intervals)
☐	☒	For null hypothesis testing, the test statistic (e.g. F, t, r) with confidence intervals, effect sizes, degrees of freedom and P value noted<br>*Give P values as exact values whenever suitable.*
☒	☐	For Bayesian analysis, information on the choice of priors and Markov chain Monte Carlo settings
☒	☐	For hierarchical and complex designs, identification of the appropriate level for tests and full reporting of outcomes
☐	☒	Estimates of effect sizes (e.g. Cohen's d, Pearson's r), indicating how they were calculated

Our web collection on statistics for biologists contains articles on many of the points above.

Software and code

Policy information about availability of computer code

Data collection	All fMRI data were collected at the Stanford Center for Cognitive and Neurobiological Imaging on a GE Discovery MR750 3T scanner using a Nova Medical 32-channel head coil at baseline and at the three drug visits. All questionnaire data were collected using REDCap version 9.3.5 through 12.5.13 on a MacBook laptop.
Data analysis	Data analysis code is available here: https://github.com/WilliamsPanLab/Ketamine-FEET-Mediation. The following preprocessing and analysis tools and packages were used: fMRIPrep version 20.2.3, SPM8, R version 4.0.5 with the mediation, lmer, and effectsize packages.

For manuscripts utilizing custom algorithms or software that are central to the research but not yet described in published literature, software must be made available to editors and reviewers. We strongly encourage code deposition in a community repository (e.g. GitHub). See the Nature Portfolio guidelines for submitting code & software for further information.

Data

Policy information about availability of data

All manuscripts must include a data availability statement. This statement should provide the following information, where applicable:

- Accession codes, unique identifiers, or web links for publicly available datasets
- A description of any restrictions on data availability
- For clinical datasets or third party data, please ensure that the statement adheres to our policy

The data used to generate figures and tables in this manuscript are available at https://github.com/WilliamsPanLab/Ketamine-FEET-Mediation.

Human research participants

Policy information about studies involving human research participants and Sex and Gender in Research.

Reporting on sex and gender	We aimed to and were able to recruit equal numbers of males and females to control for the biological sex of participants. We included biological sex as a confounder for linear mixed models that were used to examine ketamine-induced dose-dependent effects. However, we do acknowledge with our current sample size we might be under-powered to examine sex differences in the acute impact of ketamine on human neural circuit function.
Population characteristics	Detailed inclusion and exclusion criteria can be found in Suppl. Table 4. In short, we recruited from the community 13 nonclinical adult participants aged 18 to 55 years (mean = 33 years, SD = 9.82 years), with an equivalent distribution of biological sex (female: 54%, male: 46%). All participants passed the study screening procedure, reported ≥2 prior uses of ketamine, and endorsed minimal clinical symptoms at baseline consistent with inclusion and exclusion criteria.
Recruitment	Participants were recruited through Facebook Ads using Institutional Review Board-approved material. Individuals who expressed interest in the study were directed to an online screening survey in REDCap. Individuals who were eligible to participate were contacted by a research coordinator for a telephone screening. On this telephone call, research coordinators provided the individuals with additional information about the study, obtained informed consent, collected additional demographic information, and scheduled an in-person screening visit at a research clinic. All minority subjects who fulfill the inclusion criteria were eligible to participate. For this outreach, we placed advertisements in print, online media, and locations with high minority-group representation or readership, and successfully recruited a representative sample of minority subjects.
Ethics oversight	Stanford Institutional Review Board (IRB)

Note that full information on the approval of the study protocol must also be provided in the manuscript.

Field-specific reporting

Please select the one below that is the best fit for your research. If you are not sure, read the appropriate sections before making your selection.

☒ Life sciences ☐ Behavioural & social sciences ☐ Ecological, evolutionary & environmental sciences

For a reference copy of the document with all sections, see nature.com/documents/nr-reporting-summary-flat.pdf

Life sciences study design

All studies must disclose on these points even when the disclosure is negative.

Sample size	Based on our power calculation, a minimum of 5 subjects with 1 baseline session and 3 drug sessions (N = 20 sessions) would enable us to detect ketamine-induced dose-dependent effects of a similar effect size as reported in recent ketamine studies. In the current study of data collection wave 1, we collected N=13 subjects that were repeatedly measured at baseline and 3 drug sessions (N = 37 sessions, excluding baseline session).
Data exclusions	We applied a pre-established data exclusion criteria. Participants' data were excluded if more than 25% (37/148) of time points were detected as motion spikes. Volumes with frame-wise displacement >0.5 mm or std DVARS >1.5 are defined as motion spikes. One participant's data for the 0.05 mg/kg was excluded.
Replication	We are seeking renewal of the current funding that supported this project to collect wave 2 data to replicate our current findings.
Randomization	Given the within-participants design of the study, each participant received all three of the specified doses (saline, 0.05mg/kg ketamine, or 0.5mg/kg ketamine) across the duration of the trial, in a randomized order.
Blinding	The study design is double blind. Both the participants and the research team, including study clinicians, are blinded to the dose assignment. Blinded research staff includes clinical research coordinators, study clinicians, research nurses, and scanning personnel. All data collection was conducted by the blinded research team. The data analyst for interim and final analysis was unblinded, which was necessary in order for the analyst to conduct dose-dependent analyses.

Reporting for specific materials, systems and methods

We require information from authors about some types of materials, experimental systems and methods used in many studies. Here, indicate whether each material, system or method listed is relevant to your study. If you are not sure if a list item applies to your research, read the appropriate section before selecting a response.

Materials & experimental systems

n/a	Involved in the study
☒	☐ Antibodies
☒	☐ Eukaryotic cell lines
☒	☐ Palaeontology and archaeology
☒	☐ Animals and other organisms
☐	☒ Clinical data
☒	☐ Dual use research of concern

Methods

n/a	Involved in the study
☒	☐ ChIP-seq
☒	☐ Flow cytometry
☐	☒ MRI-based neuroimaging

Clinical data

Policy information about clinical studies

All manuscripts should comply with the ICMJE guidelines for publication of clinical research and a completed CONSORT checklist must be included with all submissions.

Clinical trial registration: https://clinicaltrials.gov/ct2/show/NCT03475277

Study protocol: The full internal study protocol will be shared upon reasonable request.

Data collection: Data were collected between August 2019 to November 2022. Data were collected at Stanford University in Stanford, CA. Eligibility surveys were collected through REDCap in the participants' native environment. Screening visits were conducted at the Stanford Clinical and Translational Research Unit (CTRU), a research clinic located on the Stanford Campus. Baseline visits was conducted at the Stanford Center for Cognitive and Neurobiological Imaging (CNI), a human magnetic resonance imaging (MRI) facility dedicated to research. Infusion session visits were conducted at CNI. Screening procedures were conducted by trained medical professionals including phlebotomists (blood draw), research nurses (ECG and vital signs), trained research coordinators (drug and psychiatric histories), and licensed study clinicians (physical examinations). Baseline and infusion session procedures were conducted by trained research coordinators, research nurses (IV placement), and licensed study clinicians (ketamine infusions, monitoring, and safety assessments). Participants were compensated for their participation.

Outcomes: The full list of primary and secondary outcomes can be found on clinicaltrials.gov (https://clinicaltrials.gov/ct2/show/NCT03475277). The primary outcome was predefined as dose-dependent effects of ketamine on brain activity in response to emotional expressions. This was measured using fMRI during the Facial Expression of Emotion Task (FEET). We conducted a one-way repeated Analysis of Variance in SPM — with dose as the within-participant factor — on the activation maps for threat faces (consisting of both anger and fear faces) relative to neutral faces, and happy faces relative to neutral faces. In this paper, we focused on the secondary outcomes of dose-dependent effects of ketamine on sub-components of dissociation and other altered states of consciousness. These were measured using the Clinician-Administered Dissociative States Scale (CADSS) and the 5-Dimensional Altered States of Consciousness (5D-ASC) rating scale. We used linear mixed effects models (LMMs) with dose (placebo, 0.05mg/kg or 0.5mg/kg) as the fixed effect and participant as a random effect. Finally, we tested whether specific aspects of ketamine-induced dissociation and other ASCs mediate the effect of dose on acute changes in neural activity during emotional processing by utilizing the Averaged Causal Mediation Effect (ACME) mediation model.

Magnetic resonance imaging

Experimental design

Design type: block-design task fMRI

Design specifications: In a Facial Expression of Emotion Task (FEET), a total of 240 masked emotional faces from 6 different emotions were presented nonconsciously, with 8 faces of the same emotion forming a block and each emotion block repeated for 5 times in a pseudorandom order. Each face was presented for 16.7ms (1 monitor frame), followed immediately by a neutral face perceptual mask for 150.3ms (9 monitor frames) and an interstimulus interval of 1083ms. Neutral masked faces stimuli were offset slightly by 1 degree in random directions to control for the possible detection of emotions based on perceptual features (e.g., the apparent motion in the pairing of a fear face with upraised eyebrows followed by a neutral face mask compared with the pairing of an angry face with contracted eyebrows followed by a neutral face mask).The total scan time for this fMRI task is 5:08. This task was repeated for baseline and all 3 drug sessions.

Behavioral performance measures: Participants were instructed to press a button when seeing a face, and we controlled for active attention by monitoring alertness with an eye tracking system.

Acquisition

Imaging type(s): functional and structural

Field strength: 3T

Sequence & imaging parameters: Structural Magnetic Resonance Imaging (MRI)
A T1-weighted and a T2-weighted anatomical MRI image were collected at the baseline visit. The T1-weighted MRI was collected in sagittal orientation with TR = 3.0 s, TE = 3.828 ms, FA =8, acquisition time = 8:33, field of view (FOV) = 256 × 256 mm, 3D matrix size = 320 × 320 × 230, voxel size = 0.8 mm isotropic, motion correction = PROMO. The T2-weighted

MRI was collected in sagittal orientation with TR = 2.5 s, TE = Maximum, FA = 90, acquisition time = 5:42, FOV = 240 × 240 mm, 3D matrix size = 320 × 320 × 216, voxel size = 0.8 mm isotropic, motion correction = PROMO.

Functional MRI
BOLD fMRI was acquired using the Gradient Echo sequence with axial slices in an interleaved order, TR = 2s, TE = 27.5ms, FA = 77°, acquisition time = 5:08, FOV = 222 x 222 mm, voxel size = 3 mm isotropic.

Area of acquisition: A whole-brain scan was used.

Diffusion MRI: ☐ Used ☒ Not used

Preprocessing

Preprocessing software

Results included in this paper come from preprocessing performed using fMRIPrep 20.2.3 (RRID:SCR_016216), which is based on Nipype 1.6.1; RRID:SCR_002502).

Anatomical Data Preprocessing
The T1-weighted (T1w) image was corrected for intensity non-uniformity (INU) with N4BiasFieldCorrection, distributed with ANTs 2.3.3 (RRID:SCR_004757), and used as T1w-reference throughout the workflow. The T1w-reference was then skull-stripped with a Nipype implementation of the antsBrainExtraction.sh workflow (from ANTs), using OASIS30ANTs as target template. Brain tissue segmentation of cerebrospinal fluid (CSF), white-matter (WM) and gray-matter (GM) was performed on the brain-extracted T1w using fast (FSL 5.0.9, RRID:SCR_002823). Brain surfaces were reconstructed using recon-all (FreeSurfer 6.0.1, RRID:SCR_001847) and the brain mask estimated previously was refined with a custom variation of the method to reconcile ANTs-derived and FreeSurfer-derived segmentations of the cortical gray-matter of Mindboggle (RRID:SCR_002438).

Functional Data Preprocessing
For each of the BOLD runs found per subject (across all tasks and sessions), the following preprocessing was performed. First, a reference volume and its skull-stripped version were generated by aligning and averaging 1 single-band references (SBRefs). Susceptibility distortion correction (SDC) was omitted. The BOLD reference was then co-registered to the T1w reference using bbregister (FreeSurfer) which implements boundary-based registration. Co-registration was configured with six degrees of freedom. Head-motion parameters with respect to the BOLD reference (transformation matrices, and six corresponding rotation and translation parameters) are estimated before any spatiotemporal filtering using mcflirt (FSL 5.0.9). Spatial smoothing with an isotropic, Gaussian kernel of 6mm FWHM (full-width half-maximum) was conducted before independent component analysis (ICA-AROMA).

Normalization

Volume-based spatial normalization of anatomical T1-weighted (T1w) images to standard spaces was performed through nonlinear registration with antsRegistration (ANTs 2.3.3), using brain-extracted versions of both T1w reference and the T1w template. The BOLD time-series were resampled onto their original, native space by applying the transforms to correct for head-motion. The BOLD time-series were resampled into the MNI standard space.

Normalization template

FSL's MNI ICBM 152 non-linear 6th Generation Asymmetric Average Brain Stereotaxic Registration Model [RRID:SCR_002823; TemplateFlow ID: MNI152Nlin6Asym].

Noise and artifact removal

Automatic removal of motion artifacts using ICA-AROMA was performed on the preprocessed BOLD on MNI space time-series after removal of non-steady state volumes and spatial smoothing with an isotropic, Gaussian kernel of 6mm FWHM (full-width half-maximum). Corresponding "non-aggressively" denoised runs were produced after such smoothing. FD was computed using two formulations following Power (absolute sum of relative motions) and Jenkinson (relative root mean square displacement between affines). FD and DVARS are calculated for each functional run, both using their implementations in Nipype.

Volume censoring

Frames that exceeded a threshold of 0.5 mm FD or 1.5 standardised DVARS were annotated as motion spikes. These motion spikes were included in the first-level GLM as confounders. Participants' data were excluded if more than 25% (37/148) of time points were detected as motion spikes.

Statistical modeling & inference

Model type and settings

Preprocessed fMRI data were entered into a general linear model at the individual level using SPM8 (https://www.fil.ion.ucl.ac.uk/spm/software/spm8/). Each block of emotional expressions was convolved with a canonical hemodynamic response function, and the blocks were used as regressors in the general linear model, as were motion spikes. Activation maps for threat (fear and anger facial expressions) relative to neutral faces, and for happy relative to neutral faces, were estimated to examine ketamine-induced brain activity change in response to negative and positive emotions.

Effect(s) tested

To examine our second objective to test the dose-dependent effects of ketamine on brain activity in response to emotional expressions, we conducted a one-way repeated Analysis of Variance (ANOVA) in SPM8 — with dose as the within-participant factor — on the activation maps for threat faces (consisting of both anger and fear faces) relative to neutral faces, and happy faces relative to neutral faces using a within-region (amygdala, anterior insula, and anterior cingulate cortex) voxel-wise analyses.

Specify type of analysis: ☐ Whole brain ☒ ROI-based ☐ Both

Anatomical location(s)	As established in our previous work, we defined ROIs with an automated meta-analysis approach using neurosynth.org. Specifically, we used Neurosynth uniformity (previously called forwardinference) maps with a false discovery rate (FDR) threshold of .01 for the search terms of anterior insula, amygdala, subgenual anterior cingulate cortex (sgACC) and dorsal anterior cingulate cortex (dACC). We imposed a restriction that the peak of the ROIs should have a minimum z-score of 6. For the anterior insula, we also excluded voxels with a z-score <5 to keep only the most relevant voxels spatially located in the anterior portion of the insula via visual inspection. For the amygdala, neurosynth maps were restricted by anatomically defined boundaries from the Automated Anatomical Labeling atlas.
Statistic type for inference (See Eklund et al. 2016)	Based on the pre-specified primary focus of targets, we constrained our voxel-wise ANOVA analysis using masks consisting of bilateral anterior insula, amygdala, sgACC and dACC. Conducting within-region voxel-wise analyses instead of deriving an average value per ROI enabled us to focus on the ROIs while still obtaining precision in detecting which part within the region is showing an effect.
Correction	To correct for multiple comparisons, a voxel threshold of $p < 0.001$ and a Gaussian random field theory (GRF) family-wise error (FWE) cluster-level correction at $p < 0.05$ was applied.

Models & analysis

n/a	Involved in the study
☒	☐ Functional and/or effective connectivity
☒	☐ Graph analysis
☒	☐ Multivariate modeling or predictive analysis
